# Impact of Boric Acid on the Stability of Hydrogen Peroxide in Aqueous Solution

**DOI:** 10.1021/acsomega.6c03206

**Published:** 2026-06-19

**Authors:** Fredrik Petersson, Vittorio Longo, Mats Jonsson

**Affiliations:** Department of Chemistry, 426712 KTH Royal Institute of Technology, SE − 100 44 Stockholm, Sweden

## Abstract

The reactivity of H_2_O_2_ is of importance for its role as an oxidant in many processes. It is an important oxidant produced radiolytically in nuclear reactors, and knowing its stability is of key importance when predicting corrosion of fuel cladding and structural materials. Boric acid is commonly used as pH buffer in many systems, and in pressurized water reactors, large amounts are also added to the coolant to control the fission. In general, boric acid has long been assumed to have a minor impact on the radiation chemistry of aqueous systems. In this work, we have studied how boric acid impacts heterogeneous catalytic decomposition, thermal decomposition and radiation-induced decomposition of H_2_O_2_. ZrO_2_ was used as a heterogeneous catalyst. The presence of boric acid slowed down the decomposition of H_2_O_2_ on ZrO_2_ by competing for adsorption sites on the catalyst surface. For the two homogeneous cases, the effect was the opposite; boric acid increased the rate of H_2_O_2_ decomposition. This is attributed to the formation of peroxoborate in various forms from complexation of H_2_O_2_ and boric acid. The rationale for this is suggested to be enhanced reactivity of peroxoborate toward superoxide resulting in a more efficient chain-reaction driving the peroxide decomposition.

## Graphical abstract

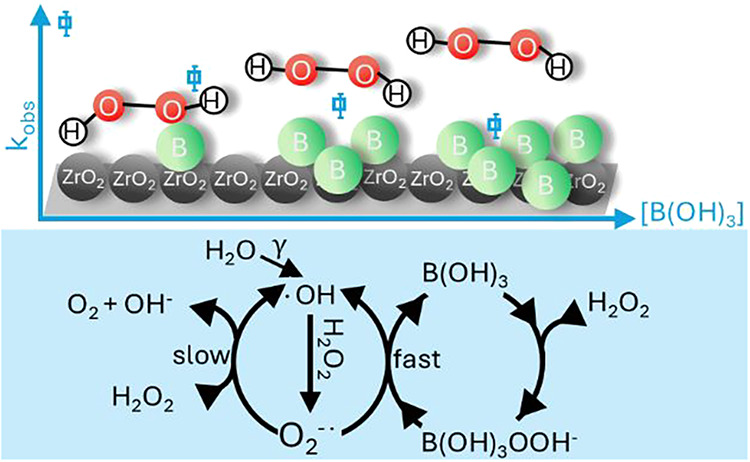

## Introduction

Hydrogen peroxide is a strong and environmentally friendly oxidant used in many household and personal care products.^1,2^ It is also used as a source of hydroxyl radicals in advanced oxidation processes, AOP, as a high efficiency, low cost and environmentally friendly way to treat wastewater.^3–5^ Another emerging use of hydrogen peroxide is as a green oxidant in organocatalytic oxidation.^6^ Industrially it is produced via the auto oxidation of anthraquinone process.^7^ It may also be produced via heterogeneous photocatalysis and as a primary product during radiolysis of water.^8–10^ Hydrogen peroxide is thermodynamically unstable but kinetically stable in aqueous solutions at room temperature.^1,11^ The rate of decomposition is strongly dependent on temperature, pH and presence of a catalyst or reactive substrate. Catalysts may be homogeneous in the form of metal ions, such as Fe^2+^, as in Fenton reactions or heterogeneous catalysts in the form of metal oxides or metals.^12–16^

The inevitable formation of hydrogen peroxide and other oxidants from radiolysis of water in light water-cooled nuclear reactors causes problems. In the same way as the oxidants can be used for treating water, they may also oxidize the fuel cladding and structural materials in the reactor. The oxides thus formed act as catalysts for H_2_O_2_ decomposition into O_2_ and H_2_O. However, homogeneous decomposition of H_2_O_2_ into O_2_ and H_2_O is also accelerated by the high temperature in nuclear reactors. In addition, the water radiolysis forming hydrogen peroxide in the reactor will contribute to its decomposition through reactions with other radiolysis products such as the hydroxyl radical (^•^OH) and the hydrated electron (e_aq_^–^).

Heterogeneous decomposition of hydrogen peroxide starts with the adsorption of hydrogen peroxide on the surface of the metal oxide. Adsorbed on the surface, the O–O bond is cleaved, and two surface-bound hydroxyl radicals, ^•^OH_ads_, are formed (reaction 1).^14^ The activation energy for homolysis of the O–O bond on the metal oxide surface is reduced significantly compared to the uncatalyzed reaction in solution.^15^ Surface-bound hydroxyl radicals can react with hydrogen peroxide, forming hydroperoxyl radicals, HO_2_^•^ (reaction 2). HO_2_^•^ can disproportionate and form H_2_O_2_ and O_2_ (reaction 3). The total reaction for surface-catalyzed decomposition of H_2_O_2_ is given by reaction 4.^17^ For metal oxides where the metal is not in its highest oxidation state, the formed ^•^OH_ads_ may also be able to oxidize the metal further. In such cases, surface oxidation will compete with catalytic decomposition of H_2_O_2_.^18,19^ Thermal homogeneous decomposition of hydrogen peroxide in aqueous solution will proceed with similar reactions.^20^

H2O2→MO2OHads• 1

H2O2+OHads•→HO2•+H2O 2

$${\mathrm{HO}}_{2^{•}}+{{\mathrm{HO}}_{2}}^{•}\to O_{2}+H_{2}O_{2}$$ 3

4 $$H_{2}O_{2}\to H_{2}O+1/2O_{2}$$

In pressurized water reactors, boric acid is added to capture thermal neutrons and thereby reduce the fission rate for freshly loaded nuclear fuel.^21^ Boric acid is also added to spent fuel storage pools to fully prevent further fission.^22^ Commonly, the boric acid used in these applications is enriched in Boron-10 due to its high cross-section for thermal neutrons. Boric acid has a p*K*_a_ of 9.23 and forms borate as a counter base.^23^ At boron concentrations exceeding 25 mM, boric acid and its counter base can start to form polyanions of di, tri and tetra borate.^24,25^ The polyanions make the boric acid/borate system more complex due to the presence of more species. It was recently shown that boric acid and borate may react with the radiolytically formed hydroxyl radical, which could have an impact on the overall radiation chemistry in systems where ^•^OH is produced in the presence of boric acid/borate.^26,27^

It is also known that boric acid or borate forms complexes with hydrogen peroxide in the form of peroxoborate.^28^ Most of the complexes start forming above pH 7 until the pH becomes high enough and H_2_O_2_ is deprotonated, p*K*_a_ 11.65.^29^ At low pH, the formation of complexes is not favored. However, one complex has a large enough formation constant so that it may be present to around 1% of peroxide in solution, that is, the peroxoboric acid, HOOB­(OH)_2_ with a formation constant, K_BO_ of 0.01 M^–1^. Peroxoboric acid forms according to reaction 5.^28^ The predominant species between pH 7 and 13 is mono- and diperoxoborates, HOOB­(OH)_3_^–^ and (HOO)_2_B­(OH)_2_^–^ respectively, which form according to reactions 6 and 7 with formation constants K_BOOH_ and K_B(OOH)2_ of 2.0 × 10^–8^ and 2.0 M^–1^, respectively.^28^ The equilibrium will be shifted toward diperoxoborate when increasing the H_2_O_2_ to B­(OH)_3_ ratio. At higher pH and at a low H_2_O_2_ to B­(OH)_3_ ratio, monoperoxodiborate, (HO)_3_BOOB­(OH)_3_^2–^ may also form according to reaction 8 with an equilibrium constant, K_BOOB_ of 4.3 M^–1^.^30^

5 $$B{\left(\mathrm{OH}\right)}_{3}+H_{2}O_{2}↔\mathrm{HOOB}{\left(\mathrm{OH}\right)}_{2}+H_{2}O$$

6 $$B{\left(\mathrm{OH}\right)}_{3}+H_{2}O_{2}↔\mathrm{HOOB}{{\left(\mathrm{OH}\right)}_{3}}^{-}+H^{+}$$

7 $$\mathrm{HOOB}{{\left(\mathrm{OH}\right)}_{3}}^{-}+H_{2}O_{2}↔{\left(\mathrm{HOO}\right)}_{2}B{{\left(\mathrm{OH}\right)}_{2}}^{-}+H_{2}O$$

8 $$\mathrm{HOOB}{{\left(\mathrm{OH}\right)}_{3}}^{-}+B{{\left(\mathrm{OH}\right)}_{4}}^{-}↔{\left(\mathrm{HO}\right)}_{3}\mathrm{BOOB}{{\left(\mathrm{OH}\right)}_{3}}^{2-}+H_{2}O$$

In addition to the formation of peroxoborates, the fact that boric acid can react with hydroxyl radicals raises the question of how it impacts the decomposition of hydrogen peroxide, since hydroxyl radicals are formed during the decomposition. It may be expected that boric acid reduces the rate of homogeneous decomposition of hydrogen peroxide due to boric acid competing for the hydroxyl radicals. The formation of peroxoborates also complicates the system, and it has previously been shown that boric acid may catalyze reactions involving hydrogen peroxide via the formation of peroxoborates.^31–33^ In this work, we present experimental data that show how boric acid affects the stability of hydrogen peroxide. ZrO_2_ is used as a heterogeneous catalyst for the catalytic decomposition of hydrogen peroxide. Homogeneous decomposition was investigated by thermal cleavage of the O–O bond at 95 °C or via γ radiolysis of water.

## Methods

All solutions were prepared with water from a Milli-Q Millipore system. Boric acid was of ACS reagent grade from Sigma-Aldrich, and Sodium hydroxide was of analytical grade from Merck. A standard solution of hydrogen peroxide from Merck with a concentration of 30% was used in all experiments. Determination of hydrogen peroxide concentration was done using the Ghormley tri-iodide method.^8,34^

Three types of experiments were performed to investigate the effect of boric acid on the stability of hydrogen peroxide: Catalytic decomposition of H_2_O_2_ on ZrO_2_ powder, thermal decomposition of H_2_O_2_ at 95 °C and γ-radiolysis of water. For the three types of experiments, several concentrations of boric acid were used (0, 100, 200, and 300 mM). To compensate for the variation in pH for the solutions with different concentrations of boric acid, the pH was adjusted to 7 with NaOH. The concentration of H_2_O_2_ at the start of every experiment was roughly 1.35 mM. Glass vials with a volume of 50 mL and plastic screw caps were used, filled with 25 mL of solution at the start of the experiment. All solutions in the experiment, unless stated, were air-saturated.

Zirconium dioxide was obtained from Merck with a purity of 99% Zirconium with a particle size smaller than 5 μm and a surface area of 6.2 m^2^/g determined by Brunauer–Emmett–Teller (BET) on a Micromeritics 3Flex. 25 ml of H_2_O_2_ solution was prepared with the various concentrations of boric acid, and 0.625 g of ZrO_2_ powder was added to start the decomposition of H_2_O_2_. The experiments were performed at room temperature. The powder was kept in a suspension through a magnetic stirring bar at 1200 rpm. Samples were taken at regular intervals with a needle and a syringe and then filtered with a cellulose acetate filter with a pore size of 0.2 μm to separate the ZrO_2_ powder from the solution and stop the decomposition.

For the second type of experiment, solutions with the various concentrations of boric acid were preheated to 95 °C inside a thermostatic bath before the H_2_O_2_ was added to the solution. As soon as the H_2_O_2_ was added, the timer started, and samples were taken at regular intervals. The vials were kept inside the thermostatic bath and kept at 95 °C for the duration of the experiments.

In the third type of experiment, samples were irradiated at room temperature in a Cs-137 Gammacell 1000 Elite. Four samples were irradiated at the same time. Due to a nonuniform dose rate inside the gammacell, the samples were rotated at a speed of 30 rpm during the irradiation. The dose rate in all four sample locations with rotation was measured to 0.101 ± 0.0002 Gy/s by Fricke dosimetry.^9^ Numerical simulations of the radiolysis system were performed by solving the set of differential equations describing the chemical reactions provided by Pastina and LaVerne.^35^ The pH in the simulations was set to 7. Reactions describing the formation of peroxoborate complexes were included with a forward rate constant of 1 × 10^3^ M^–1^ s^–1^ and the reverse reaction depending on the equilibrium constant. All reactions included in the simulations can be seen in the Supporting Information.

## Results and Discussion

To explore the kinetics of catalytic decomposition of H_2_O_2_ on ZrO_2_ powder, the concentration of H_2_O_2_ was monitored as a function of time (Figure 1). It can be seen that H_2_O_2_ is consumed faster in the solution without any boron. The rate of catalytic decomposition appears to decrease with increasing concentration of boric acid.

**Figure 1:**
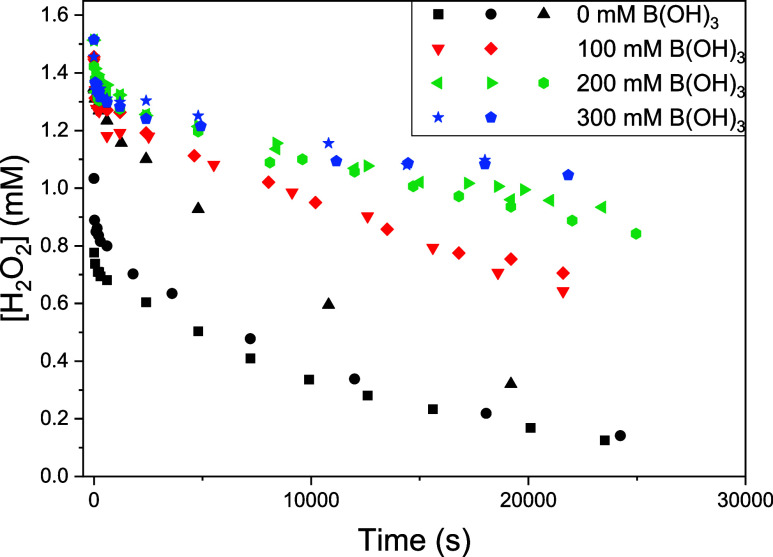
H_2_O_2_-concentration as a function of time. Black markers: Solution with 0 mM of B­(OH)_3_, Red markers: Solutions with 100 mM of B­(OH)_3_, Green markers: solution with 200 mM of B­(OH)_3_, Blue markers: solution with 300 mM of B­(OH)_3_.

It should be noted that two of the three experiments for 0 mM boric acid started at a lower concentration of H_2_O_2_. To calculate the first-order rate constant for the H_2_O_2_-decomposition, eq 9 is integrated to obtain the integrated first-order rate law, eq 10. Hence, by plotting the natural logarithm of the H_2_O_2_ concentration at time t against time, the rate constant can be obtained.

9 $$\frac{-d\left[H_{2}O_{2}\right]}{dt}=k_{\mathrm{obs}}\left[H_{2}O_{2}\right]$$

10 $$\ln\left(\frac{{\left[H_{2}O_{2}\right]}_{t}}{{\left[H_{2}O_{2}\right]}_{0}}\right)=k_{\mathrm{obs}}t$$

Admittedly, the rate of H_2_O_2_ decomposition will also depend on the surface area of ZrO_2_ in the suspension. However, if the surface area is in large excess, the reaction will follow pseudo first-order kinetics.^14^ If the surface area is small, the surface may be saturated leading to zeroth-order kinetics. In general, the kinetics of surface reactions can be described using Langmuir–Hinshelwood kinetics.^36,37^ All experiments that were performed with ZrO_2_ display first-order kinetics with respect to H_2_O_2_, and the observed first-order rate constants are seen in Figure 2. The rate constant in the absence of boric acid is determined to be 7.5 × 10^–5^ s^–1^. For the solutions with boric acid, the rate constants were determined to be less than half in the presence of 100 mM of boric acid, 3.0 × 10^–5^ s^–1^. For 300 mM of boric acid, the rate constant is 6 times lower than the rate constant without boric acid, 1.2 × 10^–5^ s^–1^.

**Figure 2:**
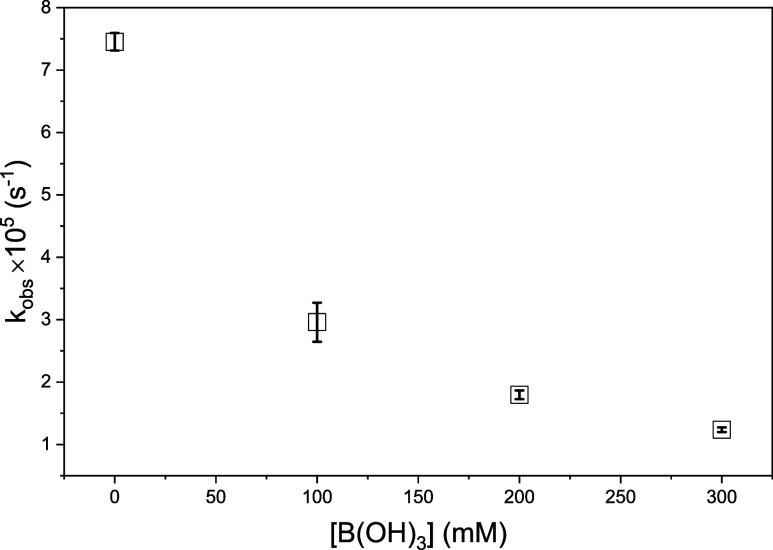
Observed first-order rate constants for the decomposition of H_2_O_2_ on the surface of ZrO_2_ as a function of B­(OH)_3_ concentration.

As can be seen, the effect of boric acid on the rate constant does not appear to be linear with concentration. One possible explanation for the observed boric acid concentration dependence on the rate of H_2_O_2_-decomposition could be adsorption of boric acid on the surface of ZrO_2_. Boric acid adsorbed on the ZrO_2_-surface could block the surface and prevent H_2_O_2_ from accessing the surface. To quantify boric acid adsorption on ZrO_2_, the concentration of boron in aqueous solution was measured with ICP-OES before and after contact with the ZrO_2_ powder. B­(OH)_3_ solutions were prepared with pH 7 and concentrations ranging from 0.5–10 mM. A low concentration had to be used in order to achieve a significant change in concentration upon adsorption. The concentration was measured after 1, 2, and 5 h, respectively, without any significant difference between the three measurements, indicating that equilibrium had been reached already before 1 h. The results are shown in Figure 3, where the fitted Langmuir isotherm is overlaid the experimental data. From the fitted Langmuir isotherm, the maximum adsorption of a monolayer corresponds to 4.13 μmol B­(OH)_3_/g ZrO_2_. At 100 mM, 98% adsorption of a monolayer will then be reached. Interestingly, the rate of H_2_O_2_ decomposition is not decreasing linearly with the surface coverage of B­(OH)_3_. In addition, the decomposition is not completely inhibited by a full monolayer of B­(OH)_3_. This implies that B­(OH)_3_ only adsorbs to a fraction of the sites responsible for the dissociative adsorption of H_2_O_2_.

**Figure 3:**
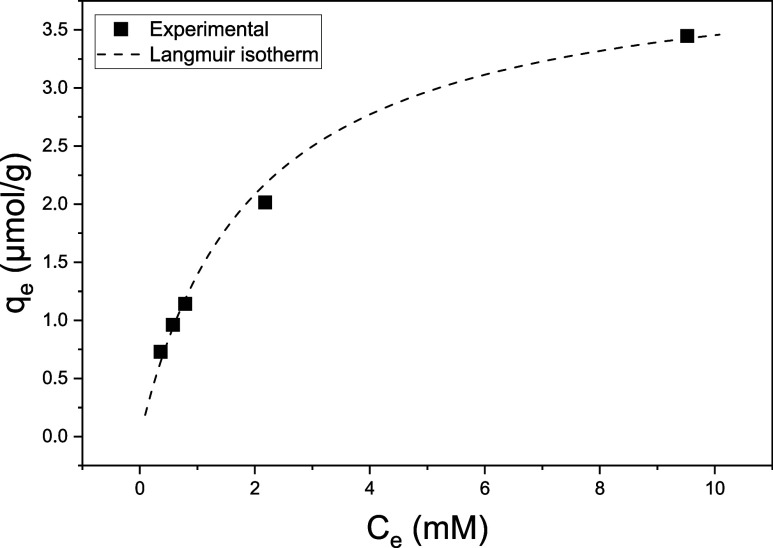
Adsorption isotherm of B­(OH)_3_ on ZrO_2_.

The effect of boric acid on the thermal stability of H_2_O_2_ in aqueous solution was investigated similarly to the experiments with ZrO_2_. In thermal decomposition, the oxygen–oxygen bond is broken homolytically in homogeneous solution. The activation energy for this process is 210 kJ mol^–1^.^38^ With an activation energy of 210 kJ mol^–1^, the H_2_O_2_ solution is supposed to be completely stable at room temperature. Any decomposition of the peroxide will be due to impurities or interactions with the surface of the storage container. To see any homolytic decomposition of H_2_O_2_, the temperature had to be increased by a large amount. In Figure 4, the concentration of H_2_O_2_ at different boric acid concentrations is plotted as a function of time at 95 °C. Interestingly, the trend with respect to boric acid concentration dependence is opposite to what was observed for catalytic decomposition of H_2_O_2_ on ZrO_2_. Addition of boric acid to the solutions increases the rate of decomposition. As mentioned above, thermal cleavage of the oxygen–oxygen bond will produce two hydroxyl radicals, which may react with H_2_O_2_, forming hydroperoxyl radicals or superoxide at pH above the p*K*_a_ of the hydroperoxyl radical.

**Figure 4:**
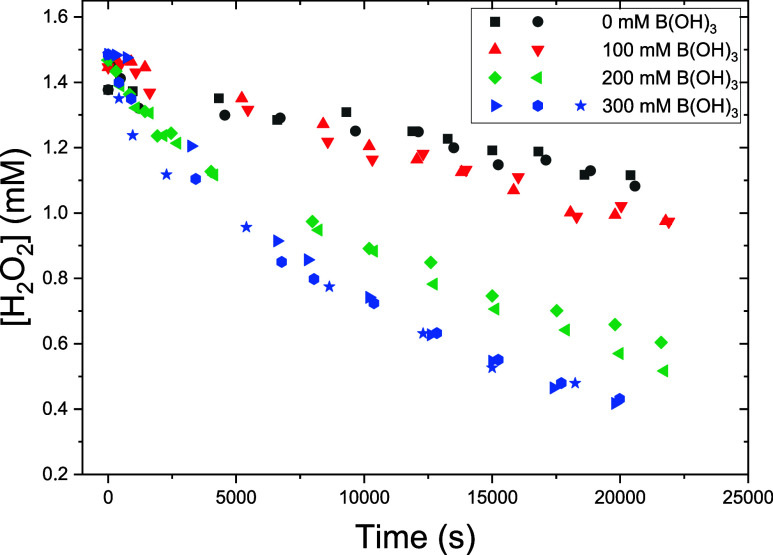
Thermal decomposition of H_2_O_2_ at 95 °C. Black markers: Solution with 0 mM of B­(OH)_3_, Red markers: Solutions with 100 mM of B­(OH)_3_, Green markers: solution with 200 mM of B­(OH)_3_, Blue markers: solution with 300 mM of B­(OH)_3_.

The first-order rate constants calculated from the data presented in Figure 4 are plotted against boric acid concentration in Figure 5. As can be seen, the rate constant increases with increasing boric acid concentration. The rationale for this trend is not straightforward. The formation of peroxodiborates, (HO)_3_BOOB­(OH)_3_^2–^ lowers the quantum yield in photolysis compared to H_2_O_2_ because the two radicals formed, ^•^OB­(OH)_3_^–^, have lower diffusivity compared to ^•^OH. This leads to more recombination of radicals and thereby a reduced quantum yield.^30^ Peroxoborate species start forming to significant extent at pH > 7.^33^ The effect that is used as rationale for the reduced quantum yield for peroxide homolysis would influence thermal homolysis in the same direction. However, this is not what we observe here. Admittedly, diffusion is temperature dependent, but we would still expect larger molecules to diffuse more slowly than smaller molecules at any given temperature. The possible rationale for the observed trend will be discussed below.

**Figure 5:**
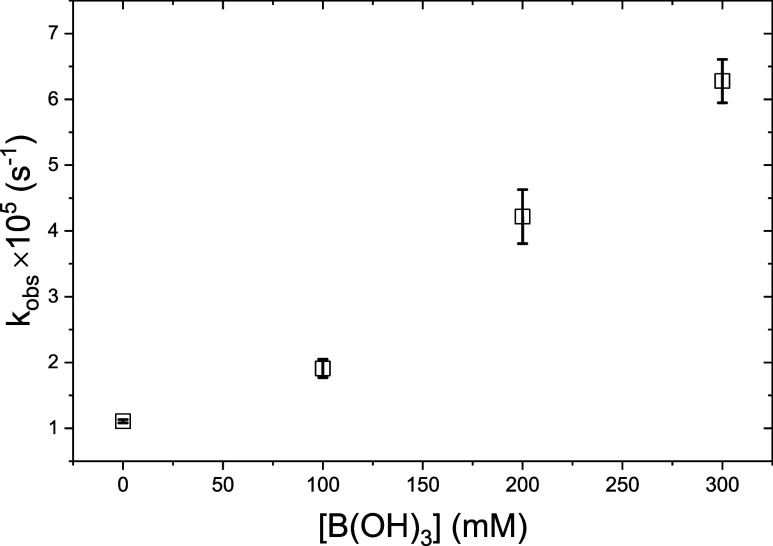
First-order rate constant for homogeneous decomposition of H_2_O_2_ at 95 °C as a function of B­(OH)_3_ concentration.

Experiments were also performed to explore the impact of boric acid on the stability of H_2_O_2_ in γ-irradiated aqueous solutions containing H_2_O_2_ from the start. The resulting H_2_O_2_-concentrations as a function of irradiation time in air-saturated aqueous solutions are illustrated in Figure 6. As can be seen, H_2_O_2_ is not completely consumed but reaches a steady-state concentration at which the rate of consumption is balanced by the rate of radiolytic production. The initial H_2_O_2_-concentration was around 1.35 mM and after 75 000 s (∼21 h), the concentration of H_2_O_2_ has reached steady-state for all four concentrations of boric acid. The final H_2_O_2_ concentration was determined as 0.15, 0.10, 0.08, and 0.06 mM for 0, 100, 200, and 300 mM of boric acid, respectively. Both the rate of H_2_O_2_-consumption (before reaching steady-state) and the final steady-state concentration follow the same pattern. The presence of boric acid increases the rate of initial decomposition, and the steady-state concentration is reduced with increasing boric acid concentration. A reduction in the steady-state concentration of H_2_O_2_ was also observed by Skotar and co-workers. In the absence of boron, a steady state concentration of H_2_O_2_ was measured to 0.08 mM and in the presence of 200 mM boron, the concentration reduced to 0.05 mM.^39^

**Figure 6:**
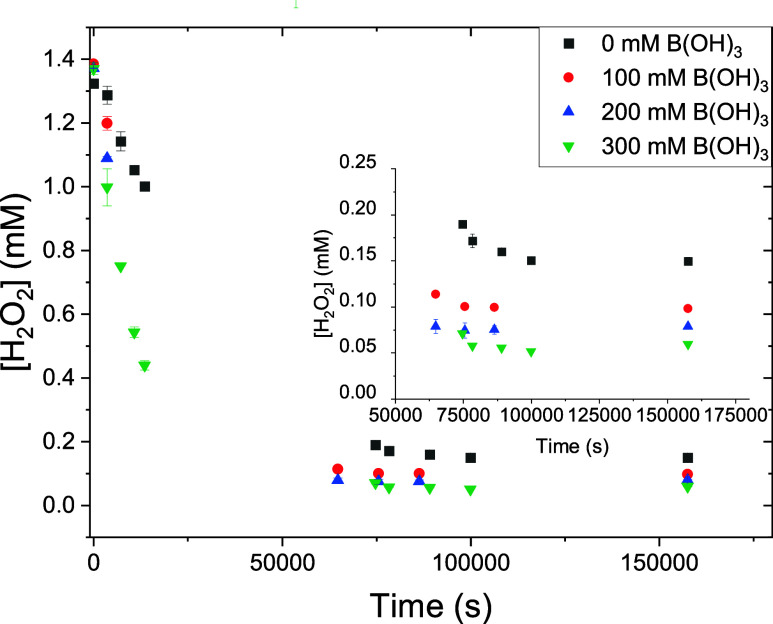
H_2_O_2_ concentration as a function of irradiation time during γ radiation with a dose rate of 0.1 Gy/s. The insert is zoomed from 50 000 s. Black markers: Solution with 0 mM of B­(OH)_3_, Red markers: Solutions with 100 mM of B­(OH)_3_, Blue markers: solution with 200 mM of B­(OH)_3_, Green markers: solution with 300 mM of B­(OH)_3_.

The results from the three experimental systems reveal two mechanistically distinct modes of action for boric acid on H_2_O_2_ stability: in the heterogeneous system, boric acid acts as an inhibitor, while in both homogeneous systems it acts as a promoter of H_2_O_2_ decomposition. The decomposition of hydrogen peroxide in γ-irradiated aqueous solution is mainly attributed to reactions with ^•^OH and e_aq_^–^ (reactions 11 and 12).^35,40^ However, in the presence of dissolved oxygen, the hydrated electron will also react with oxygen via reaction 13, forming superoxide, O_2_^•–^.^35^ The reaction between the hydroxyl radical and hydrogen peroxide at neutral pH also forms superoxide via equilibrium between HO_2_^•^ and O_2_^•–^ with a p*K*_a_ of 4.8.^41^ Hydrogen peroxide is produced in the solution as one of the primary products from water radiolysis, and the steady-state concentration strongly depends on the oxygen concentration in the water. The hydroperoxyl radical, HO_2_^•^ and superoxide may both be oxidized by hydrogen peroxide, reactions 14 and 15. However, these reactions are relatively slow compared to the disproportionation reactions, reactions 16 to 18.^42,43^

H2O2+O•H→HO2•+H2O⁣k3=2.7×107M−1s−1 11

12 H2O2+eaq−→O•H+OH−⁣k4=1.1×1010M−1s−1

$$e_{\mathrm{aq}}^{-}+O_{2}\to {O_{2}}^{•-},k_{5}=1.9\times 10^{10}M^{-1}s^{-1}$$ 13

HO2•+H2O2→O•H+O2+H2O⁣k6=0.5M−1s−1 14

15 O2•−+H2O2→O•H+O2+OH−⁣k7=0.13M−1s−1

$${{\mathrm{HO}}_{2}}^{•}+{{\mathrm{HO}}_{2}}^{•}\to O_{2}+H_{2}O_{2},k_{8}=7\times 10^{5}M^{-1}s^{-1}$$ 16

$${{\mathrm{HO}}_{2}}^{•}+{O_{2}}^{•-}\overset{(H_{2}O)}{\to }O_{2}+H_{2}O_{2}+{\mathrm{OH}}^{-},k_{9}=8\times 10^{7}M^{-1}s^{-1}$$ 17

$${O_{2}}^{•-}+{O_{2}}^{•-}\overset{2(H_{2}O)}{\to }H_{2}O_{2}+O_{2}+2{\mathrm{OH}}^{-},k_{10}=0.3M^{-1}s^{-1}$$ 18

Consumption of hydrogen peroxide under γ-irradiation was also monitored in nitrogen and oxygen-saturated solutions. The effect of 300 mM of boric acid at pH 7 was investigated. The change in hydrogen peroxide concentration as a function of time is seen in Figure 7. A set of reactions from Pastina and Laverne was used in combination with the experimentally determined dose rate and the radiation chemical yields of the radiolysis products to simulate the concentration evolution with irradiation time.^35^ In Figure 7, it is seen that the model correlates well with the experimental results for the nitrogen-saturated boron-free solution, but in the oxygen-saturated, boron-free solution, the model deviates significantly from the measured concentration. This could potentially be attributed to an incomplete model or a rate constant that has not been correctly determined. It is interesting that the oxygenated system deviates from the model. Here, the model underestimates the consumption of H_2_O_2_. The main difference between the N_2_- and O_2_-purged systems is that there will be considerable production of superoxide/hydroperoxyl radical in the O_2_-purged system. These species can contribute to the consumption of H_2_O_2_ via reactions 14 and 15. However, these reactions are very slow. The rate constants for these reactions were derived through fitting to a complex mechanism by measuring the oxygen evolution in solution as a function of H_2_O_2_.^42^ By increasing these two rate constants by 2 orders of magnitude, a better fit to the oxygen saturated solution studied in this work would be obtained.

**Figure 7:**
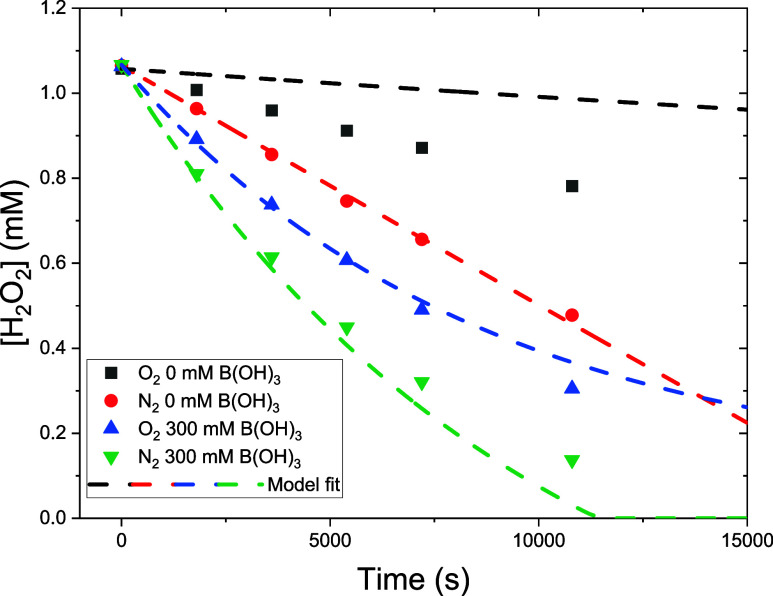
H_2_O_2_ concentration as a function of time in oxygen and nitrogen-saturated solutions without and with 300 mM of boric acid. Dashed lines are a model describing radiolytic reactions during γ radiation with a dose rate of 0.1 Gy, s^–1^.

In both oxygen and nitrogen-saturated solutions, the presence of boric acid significantly increased the rate of H_2_O_2_-consumption. As mentioned earlier, the reaction between hydrogen peroxide and the hydrated electron or the hydroxyl radicals is the main route of decomposition of hydrogen peroxide. In the nitrogen-saturated solution, more or less all of the produced electrons and hydroxyl radicals react with hydrogen peroxide initially. The rate of hydrogen peroxide consumption in nitrogen-saturated boron-free solution follows the rate of production of the hydrated electron and the hydroxyl radical to 99.5%.

The increase in the rate of H_2_O_2_-consumption in the solutions with boric acid indicates that something more than the hydroxyl radical and the hydrated electron is reacting with hydrogen peroxide. It is known that the complexes formed between boric acid or borate with hydrogen peroxide i.e., HOOB­(OH)_3_^–^, (HOO)_2_B­(OH)_2_^–^, HOOB­(OH)_2_ and (HO)_3_BOOB­(OH)_3_^2–^, increase the rate of the oxidation of both thiocyanate and organic sulfides.^31,44^ The presence of boric acid or borate can increase the rate of oxidation by hydrogen peroxide by several orders of magnitude.^31–33,44^ This is ascribed to an increased electrophilicity of the peroxoborate complexes over hydrogen peroxide.^33^ The increased electrophilicity of the peroxoborates makes them stronger oxidants than hydrogen peroxide. It is therefore reasonable to assume that peroxoborates oxidize the superoxide radical faster than hydrogen peroxide does.^45^

Hence, it is reasonable to assume that boric acid will catalyze the oxidation of the superoxide radical by hydrogen peroxide through the formation of peroxoborates. Since reaction 15 is very slow, an increase in the rate constant would significantly increase the rate of hydrogen peroxide decomposition. A reaction between superoxide and the peroxoborates forms an oxidizing radical. To model the effect of an increase in the reactivity of superoxide in the presence of the peroxoborate complexes, forward and reverse reactions are added to describe the formation of the four main peroxoborate species according to the equilibrium constants.^28,30,31^ For the purpose of the simulations, the oxidizing radical formed is written as O_3_^–^, but it could as well be a hydroxyl radical or borate radical, B­(OH)_3_O^•–^.

The oxidizing radical reacts with H_2_O_2_, oxidizing it, forming HO_2_^•^ which is deprotonated at neutral pH, forming a new superoxide radical. This leads to the formation of a chain reaction that will propagate for as long as the superoxide radical reacts with hydrogen peroxide or the peroxoborate complexes. The termination of the chain reactions will happen when the disproportionation reactions of the superoxide or hydroperoxyl radical occur, i.e., reaction 16–18, or in case ^•^OH reacts with something other than H_2_O_2_. It is difficult to determine the rate constants for the specific peroxoborate complexes since they all exist simultaneously. For the purpose of the model, all four of the reactions between the peroxoborate complexes and superoxide, were fitted with a rate constant of 3 × 10^3^ M^–1^ s^–1^. The result of this fitting can be seen as the blue and green dashed lines in Figure 7, for the nitrogen and oxygen saturated solutions containing 300 mM of boric acid. The model reproduces the trend observed experimentally with an increase in rate of decomposition of H_2_O_2_ in the presence of boric acid.

With the proposed reactions and the rate constant obtained from fitting, it is also possible to predict the steady state concentration of H_2_O_2_ with various concentrations of boric acid and compare the predictions to the experimental results. This comparison is made in Figure 8. The modeled steady-state concentration of H_2_O_2_ follows the same trend as the experimental data, with a decrease in steady-state with increasing concentration of B­(OH)_3_. However, the model underestimates the steady-state concentration with decreasing concentration of B­(OH)_3_. The reason for this is most likely due to how the evolution of gas is treated in the model. In the model, the concentration of O_2_ is fixed at the air-saturated concentration, and H_2_ is fixed at 0. In reality, the two concentrations will build up during the irradiation. However, with a headspace above the liquid it is also difficult to estimate how fast and how much the gas concentration will increase, since the headspace will slow down the buildup of O_2_ and H_2_ by allowing some to transfer from the liquid to the gas phase. Oxygen formed during the irradiation via rection 3 will scavenge the hydrated electron forming superoxide. With B­(OH)_3_ in solution, the formed superoxide can react with peroxoborate complexes leading to a smaller effect on steady-state concentration from changes in oxygen concentration. In the model, the calculated steady state concentrations in Figure 8 are closer to the experimental data when boric acid is present.

**Figure 8:**
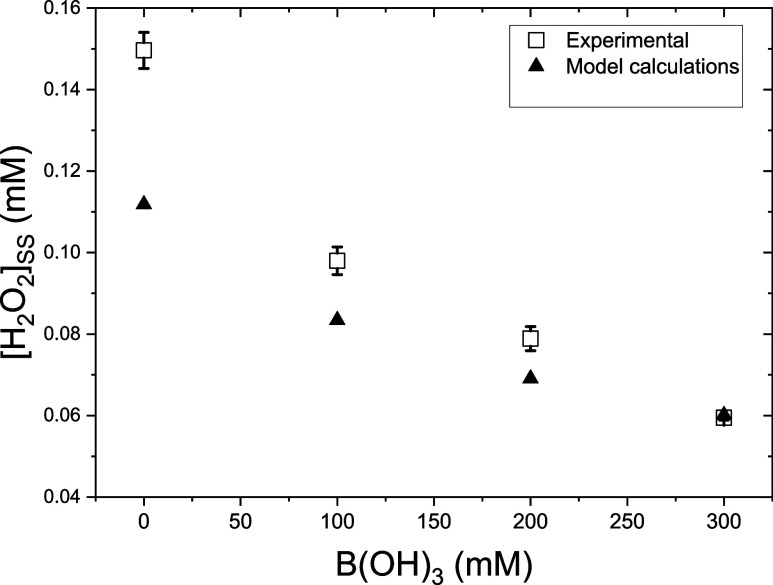
Experimentally determined steady state concentration of H_2_O_2_ during γ radiolysis as a function of boric acid concentration and the calculated steady state concentration.

Returning to the homogeneous decomposition of hydrogen peroxide at 95 °C, the explanation with the increased reactivity of superoxide toward the peroxoborate complexes as compared to H_2_O_2_ may also be applicable. The reactions following thermal homolysis of hydrogen peroxide are to a large extent the same as in an irradiated solution. Superoxide will be formed in the reaction between hydrogen peroxide and hydroxyl radicals and the superoxide may subsequently react with peroxoborate.

Based on the peroxoborate reactions discussed above we can at least qualitatively assess the impact of boric acid on the thermal decomposition of H_2_O_2_. Using the experimental rate constant for H_2_O_2_ decomposition at 95 °C without added boric acid as the basic rate constant of O–O scission and the reactions and rate constants valid at room temperature used in the radiolysis simulations, we have simulated the thermal decomposition of H_2_O_2_. The results are shown in Figure 9. As could be seen in the experiments, increasing the amount of boric acid accelerates the rate of H_2_O_2_ decomposition. Hence, increased reactivity of superoxide toward peroxoborate as compared to free hydrogen peroxide can account for the experimentally observed trend also in the case of thermal decomposition of H_2_O_2_.

**Figure 9:**
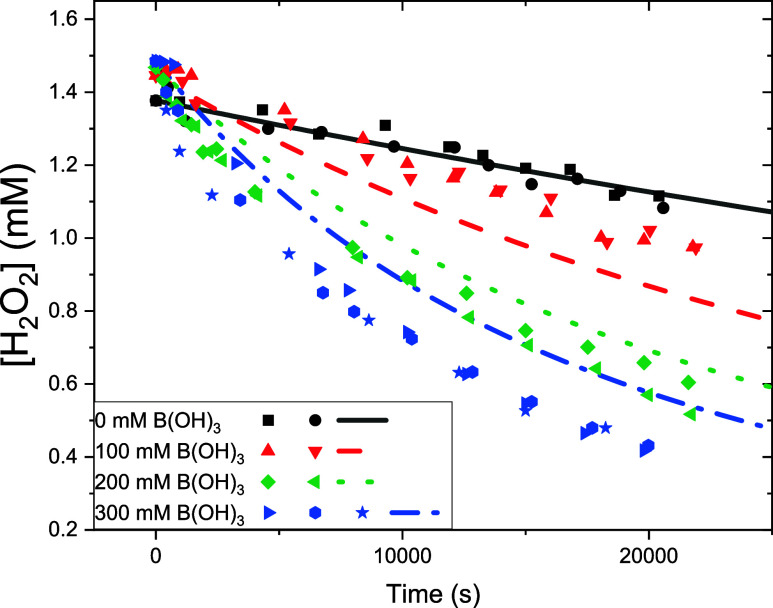
Experimentally determined concentrations of thermal decomposition of H_2_O_2_ and calculated change of H_2_O_2_ during thermal decomposition as a function of time. Black markers: Solution with 0 mM of B­(OH)_3_, Red markers: Solutions with 100 mM of B­(OH)_3_, Green markers: solution with 200 mM of B­(OH)_3_, Blue markers: solution with 300 mM of B­(OH)_3_. Lines represent the simulated concentrations.

The simulated data in Figure 9 correlate well with experimental data of the thermal homolysis. This supports the hypothesis that the rationale for the effect of boric acid in the thermal decomposition of H_2_O_2_ and radiation-induced decomposition of H_2_O_2_ in aqueous solution has the same mechanistic origin.

For the heterogeneous system, the formation of peroxoborates should also be considered in the discussion since the systems are the same except for the ZrO_2_ surface. The decrease in decomposition rate might be explained by the blocking of the surface by B­(OH)_3_ adsorption. However, the decrease in rate is not as large as expected from the adsorption isotherm of B­(OH)_3_ alone, i.e., the rate of decomposition is not proportional to the surface fraction free from B­(OH)_3_. As stated above, B­(OH)_3_ and hydrogen peroxide most probably do not compete for the same sites, although there might be some partial overlap so that adsorption of B­(OH)_3_ affects the sites available for H_2_O_2_.

The surface reactivity of peroxoborates should also be considered. Under the present experimental conditions, the majority of the hydrogen peroxide is noncomplexed, and unless the reactivity of peroxoborates is much higher, the effect will be small. However, the reactivity of peroxoborates toward ZrO_2_ is unknown. The reaction of superoxide toward the peroxoborates is also of potential importance for the surface-catalyzed decomposition. The surface-formed hydroperoxyl radicals from the hydrogen peroxide decomposition might potentially escape the surface and form superoxide instead of disproportionating to H_2_O_2_ and O_2_ on the surface. The superoxide can then react with peroxoborates, initiating the chain reaction discussed above for the radiation-induced decomposition of H_2_O_2_. As shown for the homogeneous systems, this would result in faster peroxide decomposition at higher B­(OH)_3_ concentrations. The fact that the rate of peroxide decomposition on ZrO_2_ decreases with increasing B­(OH)_3_ concentration, indicates that the effect of surface blocking is larger than the effect of increased reactivity between superoxide and peroxoborate.

## Conclusions

The results presented here show that boric acid has an impact on the rate of hydrogen peroxide decomposition in three different systems. The presence of boric acid increases the rate of hydrogen peroxide decomposition in aqueous solutions exposed to γ-radiation and in aqueous solutions at elevated temperatures. The rationale for this is argued to be increased reactivity of superoxide toward hydrogen peroxide complexed with boric acid (peroxoborate) compared to free hydrogen peroxide. A rate constant of 3 × 10^3^ M^–1^ s^–1^ for the reaction between superoxide and peroxoborate quantitatively accounts for the experimental observations of the irradiated system and semiquantitatively for the experimental observation at elevated temperature. In the heterogeneous system, hydrogen peroxide in aqueous solution is catalytically decomposed on ZrO_2_(s). In this system the presence of boric acid decreases the rate of hydrogen peroxide decomposition. Even though the increased reactivity of superoxide toward peroxoborate should have an influence also in the heterogeneous system, this effect is overshadowed by the adsorption of boric acid on the ZrO_2_-surface. This process reduces the surface area available for peroxide decomposition and thereby reduces the rate.

## Supplementary Material



## Notes

Chemical reactions; and equilibrium constants and primary yields for the radiolysis used for the simulations (PDF)
