# Supplementary material for: Impact of Boric Acid on the Stability of Hydrogen Peroxide in Aqueous Solution
Source: ACS Omega. 2026 Jun 19;11(25):37919–26. doi: 10.1021/acsomega.6c03206 (PMC13325353; doi:10.1021/acsomega.6c03206)
Supplement: Supplementary file 1 [file ao6c03206_si_001.pdf]

Impact of Boric Acid on the Stability of Hydrogen Peroxide in Aqueous Solution

Fredrik Petersson*, Vittorio Longo and Mats Jonsson, Department of Chemistry, KTH Royal Institute of Technology, SE – 100 44 Stockholm, Sweden

*Frepeter@kth.se

Content

Table S1 Equilibrium reactions and chemical reactions 1

Table S2 Reactions describing boric acid equilibria and peroxoborate equilibria and its reaction with the superoxide radical. 4

Table S3. Equilibrium constants 4

Table S4. Primary radiolysis reactions and G-Values describing the radiolytic yield. 5

Table S1. Equilibrium reactions and chemical reactions used in the simulations of the radiolysis and thermal decomposition of H_2O_2 taken from Pastina and LaVerne [35]

**Reaction no.**	**Chemical reaction**	**Rate constant (M^{-1}, s^{-1} or s^{-1})**
1	$H^+ + OH^- \rightarrow H_2O$	1.4×10^{11}
2	$H_2O \rightarrow H^+ + OH^-$	k1 * K1 / H_2O
3	$H_2O_2 \rightarrow H^+ + HO_2^-$	k4 * K2
4	$H^+ + HO_2^- \rightarrow H_2O_2$	5.0×10^{11}
5	$H_2O_2 + OH^- \rightarrow HO_2^- + H_2O$	1.3×10^{11}
6	$HO_2^- + H_2O \rightarrow H_2O_2 + OH^-$	k5 * K1 / K2 / H_2O
7	$e^-_{aq} + H_2O \rightarrow H + OH^-$	1.9×10^{1}
8	$H + OH^- \rightarrow e^-_{aq} + H_2O$	2.2×10^{7}
9	$H \rightarrow e^-_{aq} + H^+$	k10 * K6
10	$e^-_{aq} + H^+ \rightarrow H$	2.3×10^{10}
11	$\cdot OH + OH^- \rightarrow O^{\cdot-} + H_2O$	1.3×10^{10}
12	$O^{\cdot-} + H_2O \rightarrow \cdot OH + OH^-$	k11 * K1 / K3 / H_2O
13	$\cdot OH \rightarrow O^{\cdot-} + H^+$	k14 * K3
14	$O^{\cdot-} + H^+ \rightarrow OH\cdot$	1.00×10^{11}
15	$HO_2\cdot \rightarrow O_2^{\cdot-} + H^+$	k22 * K4
16	$O_2^{\cdot-} + H^+ \rightarrow HO_2\cdot$	5.00×10^{10}
17	$HO_2\cdot + OH^- \rightarrow O_2^{\cdot-} + H_2O$	5.00×10^{10}
18	$O_2^{\cdot-} + H_2O \rightarrow HO_2 + OH^-$	k17 * K1 / K4 / H_2O
19	$e^-_{aq} + \cdot OH \rightarrow OH^-$	3.0×10^{10}
20	$e^-_{aq} + H_2O_2 \rightarrow \cdot OH + OH^-$	1.1×10^{10}
21	$e^-_{aq} + O_2^{\cdot-} + H_2O \rightarrow HO_2^- + OH^-$	1.3×10^{10} / H_2O

22	$e^-_{aq} + HO_2\cdot \rightarrow HO_2^-$	2.0×10^{10}
23	$e^-_{aq} + O_2 \rightarrow O_2^{\cdot-}$	1.9×10^{10}
24	$e^-_{aq} + e^-_{aq} + 2H_2O \rightarrow H_2 + {}_2OH^-$	5.5×10^{9} / H_2O
25	$e^-_{aq} + H + H_2O \rightarrow H_2 + OH^-$	2.5×10^{10} / H_2O
26	$e^-_{aq} + HO_2^- \rightarrow O^{\cdot-} + OH^-$	3.5×10^{9}
27	$e^-_{aq} + O^{\cdot-} + H_2O \rightarrow {}_2OH^-$	2.2×10^{10} / H_2O
28	$e^-_{aq} + O_3^- + H_2O \rightarrow O_2 + {}_2OH^-$	1.6×10^{10} / H_2O
29	$e^-_{aq} + O_3\cdot \rightarrow O_3^-$	3.6×10^{10}
30	$H\cdot + H_2O \rightarrow H_2 + \cdot OH$	1.1×10^{1}
31	$H\cdot + O^{\cdot-} \rightarrow OH^-$	1.0×10^{10}
32	$H\cdot + HO_2^- \rightarrow \cdot OH + OH^-$	9.0×10^{7}
33	$H\cdot + O_3^- \rightarrow OH^- + O_2$	1.0×10^{10}
34	$H\cdot + H\cdot \rightarrow H_2$	7.8×10^{9}
35	$H\cdot + \cdot OH \rightarrow H_2O$	7.0×10^{9}
36	$H\cdot + H_2O_2 \rightarrow \cdot OH + H_2O$	9.0×10^{7}
37	$H\cdot + O_2 \rightarrow HO_2\cdot$	2.1×10^{10}
38	$H\cdot + HO_2\cdot \rightarrow H_2O_2$	1.8×10^{10}
39	$H\cdot + O_2^{\cdot-} \rightarrow HO_2^-$	1.8×10^{10}
40	$H\cdot + O_3\cdot \rightarrow HO_3\cdot$	3.8×10^{10}
41	$\cdot OH + \cdot OH \rightarrow H_2O_2$	3.6×10^{9}
42	$\cdot OH + HO_2\cdot \rightarrow H_2O + O_2$	6.0×10^{9}
43	$\cdot OH + O_2^{\cdot-} \rightarrow OH^- + O_2$	8.2×10^{9}
44	$\cdot OH + H_2 \rightarrow H + H_2O$	4.3×10^{7}
45	$\cdot OH + H_2O_2 \rightarrow HO_2\cdot + H_2O$	2.7×10^{7}
46	$\cdot OH + O^{\cdot-} \rightarrow HO_2^-$	2.5×10^{10}
47	$\cdot OH + HO_2^- \rightarrow HO_2\cdot + OH^-$	7.5×10^{9}
48	$\cdot OH + O_3^- \rightarrow O_3 + OH^-$	2.6×10^{9}
49	$\cdot OH + O_3^- \rightarrow 2O_2^- + H^+$	6.0×10^{9}
50	$\cdot OH + O_3^- \rightarrow HO_2\cdot + O_2$	1.1×10^{8}
51	$HO_2\cdot + O_2^{\cdot-} \rightarrow HO_2^- + O_2$	8.0×10^{7}
52	$HO_2\cdot + HO_2\cdot \rightarrow H_2O_2 + O_2$	7.0×10^{5}
53	$HO_2\cdot + O^{\cdot-} \rightarrow O_2 + OH^-$	6.0×10^{9}
54	$HO_2\cdot + H_2O_2 \rightarrow \cdot OH + O_2 + H_2O$	5.0×10^{-1}
55	$HO_2\cdot + HO_2^- \rightarrow \cdot OH + O_2 + OH^-$	5.0×10^{-1}
56	$HO_2\cdot + O_3^- \rightarrow {}_2O_2 + OH^-$	6.0×10^{9}
57	$HO_2\cdot + O_3\cdot \rightarrow HO_3\cdot + O_2$	5.0×10^{8}
58	$O_2^{\cdot-} + O_2^{\cdot-} + 2H_2O \rightarrow H_2O_2 + O_2 + {}_2OH^-$	0.3 / ($2\times H_2O$)
59	$O_2^{\cdot-} + O^{\cdot-} + H_2O \rightarrow O_2 + {}_2OH^-$	6.0×10^{8} / H_2O
60	$O_2^{\cdot-} + H_2O_2 \rightarrow \cdot OH + O_2 + OH^-$	1.3×10^{-1}

61	$O_2^{\cdot-} + HO_2^- \rightarrow O^{\cdot-} + O_2 + OH^-$	1.3×10^{-1}
62	$O_2^{\cdot-} + O_3^- + H_2O \rightarrow {}_2O_2 + {}_2OH^-$	1.0×10^4 / H_2O
63	$O_2^{\cdot-} + O_3\cdot \rightarrow O_3^- + O_2$	1.5×10^9
64	$O^{\cdot-} + O^{\cdot-} + H_2O \rightarrow HO_2^- + OH^-$	1.0×10^9 / H_2O
65	$O^{\cdot-} + O_2 \rightarrow O_3^-$	3.6×10^9
66	$O^{\cdot-} + H_2 \rightarrow H + OH^-$	8.0×10^7
67	$O^{\cdot-} + H_2O_2 \rightarrow O_2^{\cdot-} + H_2O$	5.0×10^8
68	$O^{\cdot-} + HO_2^- \rightarrow O_2^{\cdot-} + OH^-$	4.0×10^8
69	$O^{\cdot-} + O_3^- \rightarrow 2O_2^{\cdot-}$	7.0×10^8
70	$O^{\cdot-} + O_3\cdot \rightarrow O_2^{\cdot-} + O_2$	5.0×10^9
71	$O_3^- \rightarrow O_2 + O^{\cdot-}$	3.3×10^3
72	$O_3^- + H^+ \rightarrow O_2 + \cdot OH$	9.0×10^{10}
73	$HO_3\cdot \rightarrow O_2 + \cdot OH$	1.1×10^5

Table S2. Reactions describing boric acid equilibria, peroxoborate equilibria and its reaction with the superoxide radical.

Reaction no.	Reactions involving boric acid or borate	Rate constant (M^{-1}, s^{-1} or s^{-1})
74	$B(OH)_3 + OH^- \rightarrow B(OH)_4^-$	k75 / K6
75	$B(OH)_4^- \rightarrow B(OH)_3 + OH^-$	1.0×10^6
76	$H_2O_2 + B(OH)_3 \rightarrow HOOB(OH)_3^- + H^+$	1.0×10^3
77	$HOOB(OH)_3^- + H^+ \rightarrow H_2O_2 + B(OH)_3$	k76 / K7
78	$HOOB(OH)_3^- + H_2O_2 \rightarrow (HOO)_2B(OH)_2^- + H_2O$	1.0×10^3
79	$(HOO)_2B(OH)_2^- + H_2O \rightarrow HOOB(OH)_3^- + H_2O_2$	k78 / K8 / H_2O
80	$B(OH)_4^- + HOOB(OH)_3^- \rightarrow (HO)_3BOOB(OH)_3^{2-} + H_2O$	1.0×10^3
81	$(HO)_3BOOB(OH)_3^{2-} + H_2O \rightarrow B(OH)_4^- + HOOB(OH)_3^-$	k80 / K9 / H_2O
82	$B(OH)_3 + H_2O_2 \rightarrow HOOB(OH)_2 + H_2O$	1.0×10^3
83	$HOOB(OH)_2 + H_2O \rightarrow B(OH)_3 + H_2O_2$	k82 / K10 / H_2O
84	$HOOB(OH)_3^- + O_2^{\cdot-} \rightarrow O_3^- + B(OH)_4^-$	3.0×10^3
85	$(HO)_3BOOB(OH)_3^{2-} + O_2^{\cdot-} + H_2O \rightarrow O_3^- + 2B(OH)_4^-$	3.0×10^3 / H_2O
86	$HOOB(OH)_2 + O_2^{\cdot-} \rightarrow O_3^- + B(OH)_3$	3.0×10^3
87	$(HOO)_2B(OH)_2^- + O_2^{\cdot-} + H_2O \rightarrow O_3^- + B(OH)_4^- + H_2O_2$	3.0×10^3/ H_2O

Table S3. Equilibrium constants taken from [23, 28, 30, 35]

Equilibrium no.	Equilibrium reactions	Equilibrium constants (-log), K_n
1	$H_2O \leftrightarrow H^+ + OH_-$	13.999
2	$H_2O_2 \leftrightarrow H^+ + HO_2^-$	11.65
3	$OH \leftrightarrow H^+ + O^{\cdot-}$	11.9
4	$HO_2^{\cdot} \leftrightarrow H^+ + O2^{\cdot-}$	4.57
5	$H\cdot \leftrightarrow H^+ + e^-_{aq}$	9.77
6	$B(OH)_4^- \leftrightarrow B(OH)_3 + OH^-$	4.78
7	$H_2O_2 + B(OH)_3 \leftrightarrow HOOB(OH)_3^- + H^+$	7.70
8	$HOOB(OH)_3^- + H_2O_2 \leftrightarrow (HOO)_2B(OH)_2^- + H_2O$	-0.30
9	$B(OH)_4^- + HOOB(OH)_3^- \leftrightarrow (HO)_3BOOB(OH)_3^{2-} + H_2O$	-0.63
10	$B(OH)_3 + H_2O_2 \leftrightarrow HOOB(OH)_2 + H_2O$	2

Table S4. Primary radiolysis reactions and G-Values describing the radiolytic yield taken from Pastina and LaVerne [35]

**Radiolysis reaction no.**	**Radiolysis reactions**	**G-Value (µmol, J^{-1})**
1	$H_2O \rightarrow e^-_{aq}$	0.269
2	$H_2O \rightarrow H\cdot$	0.068
3	$H_2O \rightarrow H_2$	0.047
4	$H_2O \rightarrow \cdot OH$	0.280
5	$H_2O \rightarrow H_2O_2$	0.073
6	$H_2O \rightarrow HO_2^{\cdot}$	0.002
7	$H_2O \rightarrow H^+$	0.321
8	$H_2O \rightarrow OH^-$	0.052
